# VentrEX: An Anatomically Guided Deep Learning Pipeline for Ventricular Segmentation in Cine Cardiac MRI

**DOI:** 10.3390/jimaging12090416

**Published:** 2026-09-03

**Authors:** Abla Bedoui, Julieta Anahí Rancati, Ignacio Lugones, Mohammed Cherkaoui

**Affiliations:** 1 School of Engineering, Computer Science and Artificial Intelligence, Long Island University, Brooklyn, NY 11201, USA; mohammed.cherkaoui@liu.edu; 2 Unidad de Cirugía Cardiovascular, Hospital General de Niños “Dr. Pedro de Elizalde”, Buenos Aires C1270AAN, Argentina; julietarancati@gmail.com (J.A.R.); ignacio.lugones@liu.edu (I.L.)

**Keywords:** cardiac MRI, deep learning, transformer, ventricular segmentation, papillary muscles, trabeculae, explainable AI, domain shift

## Abstract

Automated segmentation of the left and right ventricles (LVs and RVs) in cine cardiac MRI (CMR) underpins reliable volumetry and mass estimation. However, papillary muscles and trabeculae (PM/T) introduce clinically meaningful variability and exacerbate cross-dataset domain shift. We present VentrEX, an anatomically guided pipeline. The core segmenter, VentrEX-Seg, is a 3D encoder-decoder with parallel channel-spatial attention and a Transformer bottleneck. Training is performed exclusively on ACDC. A lightweight PM/T module automatically extracts papillary and trabecular burdens and standardizes cavity volumes. External evaluation is zero-shot (no fine-tuning) on Sunnybrook (LV) and MM-WHS MRI (RV). We report Dice, HD95 (mm); for volumetry, we use Bland-Altman analyses (LV and RV volumes). Attention/Grad-CAM visualizations support interpretability. On ACDC, VentrEX achieved higher Dice and lower boundary error than U-Net, nnU-Net, CBAM, and VentrEX-Seg. Zero-shot performance was preserved externally (e.g., Sunnybrook LV Dice 0.9053, HD95 4.95 mm; MM-WHS RV Dice 0.9236, HD95 6.61 mm). Patient-level Bland–Altman analyses characterized LV and RV volumetric agreement. Qualitative overlays and 3D reconstructions showed fewer PM/T “leaks” and anatomically plausible borders across ED/ES. Single-source training with dual zero-shot external tests demonstrates robustness under domain shift. The combination of parallel attention and a Transformer bottleneck enables accurate, transparent cine-CMR segmentation across datasets.

## 1. Background

The exponential growth of cardiac imaging data driven by advances in cine cardiac MRI (CMR) acquisition and clinical adoption creates both an opportunity to extract objective quantitative phenotypes and a challenge for current analysis pipelines. Quantitative assessment of cardiac function relies on biomarkers such as LV end-diastolic/systolic volumes (LVEDV/LVESV), LV mass (LVM), LV ejection fraction (LVEF), stroke volume, and regional wall thickening. Computing any of these requires accurate ventricular segmentation. Yet manual or semi-manual contouring is time-consuming and reader-dependent; even under consensus protocols, inter-center bias has been documented [1]. Endocardial structures, particularly papillary muscles and trabeculae (PM/T), are especially consequential: alternative inclusion or exclusion can shift LVM and EF by clinically meaningful margins, undermining cross-site comparability [2].

Deep learning has reshaped CMR segmentation [3,4,5,6]. U-Net established the modern encoder-decoder paradigm with skip connections for high-resolution detail [7], and nnU-Net showed that a self-configuring recipe can achieve strong performance across diverse datasets without bespoke tuning [8]. To capture long-range context beyond local convolutions, hybrid CNN-Transformer designs such as TransUNet integrate self-attention within U-Net backbones [9]. Recent Transformer-U-Net variants have incorporated complementary attention mechanisms to improve feature localization while preserving global context. DA-TransUNet integrates spatial and channel dual-attention modules into a Transformer-U-Net architecture, whereas EG-TransUNet employs enhanced and guided feature modules for biomedical image segmentation [10,11]. For cardiac MRI specifically, hybrid CNN-Transformer architectures have also been investigated for large-scale ventricular segmentation, demonstrating their potential for robust anatomical delineation [12].

However, two practical gaps continue to limit clinical translation. (i) The first is domain shift [13]: models trained on a single source often degrade on external cohorts that differ in scanners, protocols, or label conventions (e.g., ACDC [14] vs. Sunnybrook Left Ventricle Segmentation Challenge (LVSC) [15] vs. MM-WHS MRI [16]); strong internal Dice does not guarantee cross-dataset agreement on volumetric biomarkers. (ii) The second gap is PM/T-driven volumetric bias: small endocardial discrepancies propagate to measurable shifts in LVM and EF even when overlap metrics are high [2]. In parallel, the opacity of deep models has slowed adoption; attention Grad-CAM-based visualizations are increasingly used to verify that decision-relevant regions align with anatomy [17].

Motivated by these considerations, we propose VentrEX, an anatomically guided two-stage pipeline comprising (1) ventricular segmentation using VentrEX-Seg, a 3D encoder-decoder that combines parallel channel-spatial attention across the encoder and decoder with a compact Transformer bottleneck, and (2) a cine-CMR-based PM/T standardization stage developed using cardiologist-validated annotations. The attention and Transformer components are not individually presented as novel. Rather, VentrEX differs from existing Transformer-U-Net variants through their three-dimensional integration with explicit PM/T standardization, zero-shot external evaluation, and patient-level volumetric-agreement analysis. The model is trained exclusively on ACDC and evaluated without fine-tuning on Sunnybrook for LV segmentation and the MRI subset of MM-WHS for RV segmentation. Attention-based visualizations are additionally provided to support interpretation and clinical review.

The principal contributions of this study are threefold:

1. We introduce VentrEX-Seg, a 3D residual encoder-decoder that combines parallel channel-spatial attention throughout the encoder and decoder with a compact Transformer bottleneck for joint ED/ES ventricular segmentation.
2. We develop a lightweight PM/T standardization stage that performs voxelwise harmonization of papillary-muscle and trabecular assignments and produces a convention-consistent ventricular blood-pool mask.
3. We evaluate the complete VentrEX pipeline through single-source training on ACDC and zero-shot testing on Sunnybrook and MM-WHS, complemented by patient-level Bland-Altman volumetric analysis and attention-based interpretability.

Our paper is organized as follows. Section 2 (Methods) presents the VentrEX pipeline, core segmenter, explainability approach, automated PM/T detection, and evaluation metrics. Section 3 (Results) reports internal and zero-shot external performance (DSC, HD95), Bland–Altman volumetry, and qualitative/interpretability findings. We then provide the Discussion (Section 4) and Conclusions (Section 5).

## 2. Methods

### 2.1. Pipeline Overview

**Figure 1:**
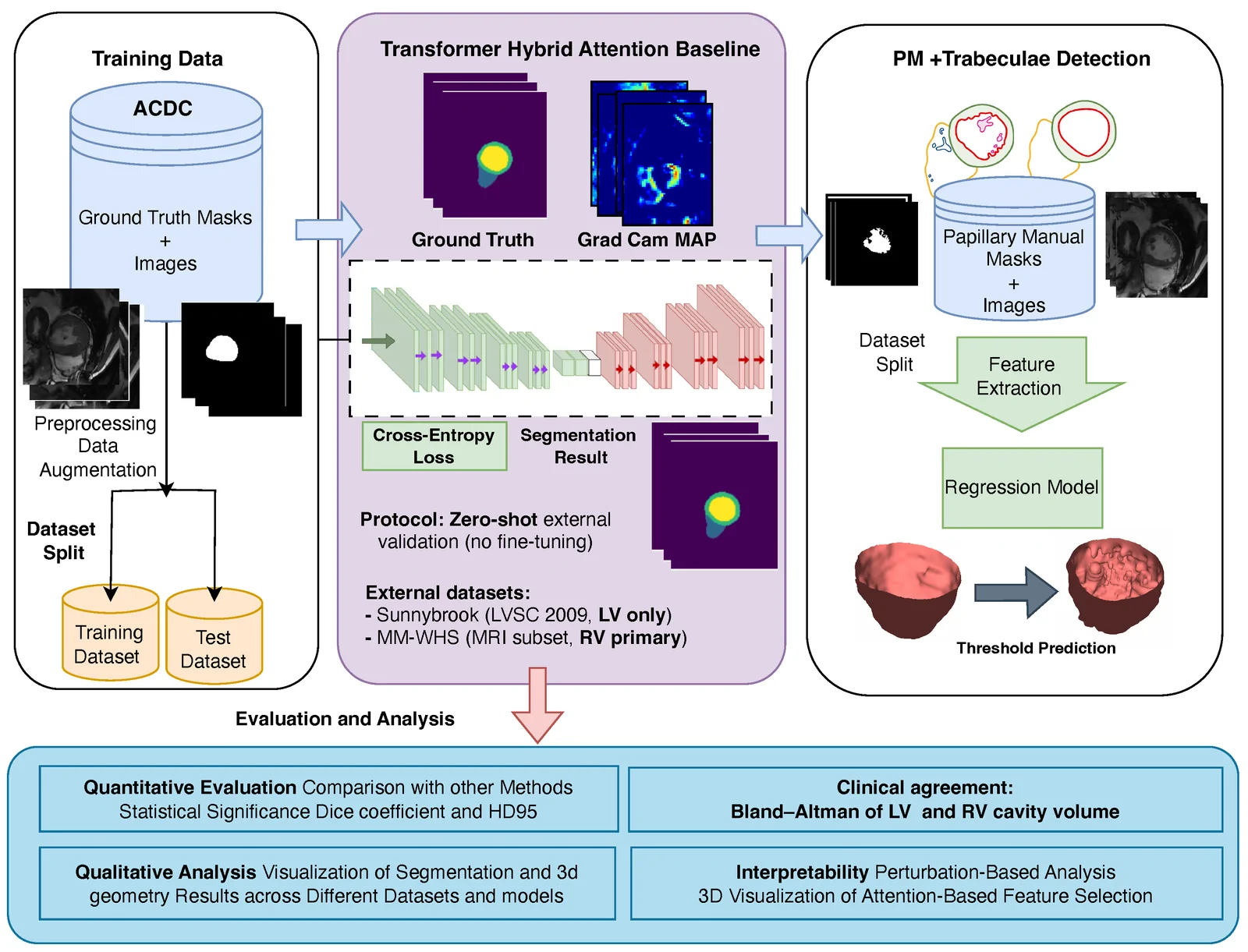
Overview of the VentrEX pipeline from preprocessing and VentrEX-Seg to PM/T standardization and evaluation. ACDC [14] was used for model development and held-out testing, whereas Sunnybrook/LVSC [15] and MM-WHS [16] were used for zero-shot external validation. Blue horizontal arrows indicate the main workflow from the input data through VentrEX-Seg to PM/T standardization. Green arrows denote feature extraction and regression-based threshold prediction, the pink downward arrow connects the pipeline outputs to the evaluation and analysis stage, and gray or black arrows indicate processing or transformation steps within individual stages.

The VentrEX pipeline comprises two stages, as depicted in Figure 1: (1) ventricular segmentation with VentrEX-Seg, which produces the primary 3D ventricular geometry, and (2) cine-based PM/T standardization, which automatically quantifies papillary muscles and trabeculae using a regression-based thresholding module. Attention and Grad-CAM visualizations support interpretability by highlighting decision-relevant regions. Data preprocessing, training protocol, and evaluation metrics are described below. Throughout this manuscript, VentrEX-Seg denotes the core three-dimensional neural segmentation network before anatomical standardization. VentrEX denotes the complete inference pipeline, comprising VentrEX-Seg followed by the adaptive PM/T standardization stage. The volumetric and attention-based explainability analyses are used to evaluate and interpret the pipeline but do not alter the predicted segmentation masks.

**Preprocessing and input preparation.** For each subject, the end-diastolic (ED) and end-systolic (ES) frames were extracted from the four-dimensional cine-CMR sequence. Each frame was center-cropped to eight short-axis slices with an in-plane size of 144×144 pixels; volumes containing fewer than eight slices were zero-padded along the through-plane dimension. The ED and ES crops were concatenated along the depth dimension to produce a per-sample input tensor with dimensions C×D×H×W=1×16×144×144, excluding the batch dimension. Intensities were standardized by subtracting the mean and dividing by the standard deviation of the cropped ED/ES input. Training augmentation comprised independent horizontal and vertical flips with a probability of 0.5; affine transformations with a probability of 0.5, using rotations of up to ±10° and scaling factors from 0.9 to 1.1; and Gaussian noise with a probability of 0.2 and a standard deviation of up to 0.05. Cubic interpolation was used for images and nearest-neighbor interpolation for segmentation labels.

No isotropic resampling was applied to the network inputs. Prediction and reference masks were resampled to an isotropic spacing of 1.25 mm only for surface-distance evaluation, whereas volumetric measurements were calculated using the physical voxel spacing stored in the corresponding image metadata. The same spatial preprocessing, cropping or padding, and intensity-standardization procedures were applied to the ACDC, Sunnybrook, and MM-WHS datasets.

### 2.2. Core Segmenter (VentrEX-Seg)

**Figure 2:**
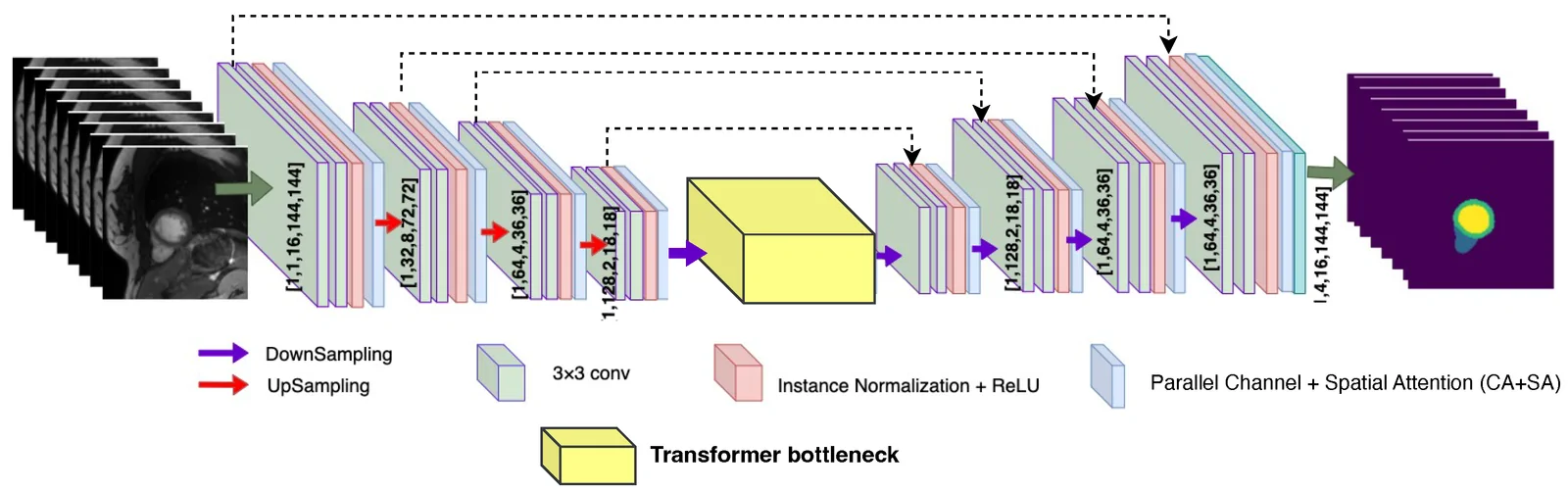
VentrEX-Seg architecture. 3D encoder-decoder with parallel channel-spatial attention at each scale and a Transformer bottleneck; decoder concatenates skips and a 1 × 1 × 1 head outputs BG, RV, MYO, and LV.

The core of the pipeline is VentrEX-Seg, a 3D encoder-decoder tailored to cine-CMR segmentation (Figure 2). The encoder stacks residual blocks with instance normalization and inserts, at each scale, parallel channel-spatial attention to highlight cardiac structures while suppressing background.

The decoder restores resolution via transpose-convolution UpBlocks, concatenates the corresponding encoder features through skip connections, and applies additional residual + parallel-attention blocks for feature selection. A final convolution with softmax assigns each voxel to four classes: background, right ventricle, myocardium, and left ventricle. VentrEX-Seg integrates three main innovations:

- **Parallel attention:** channel and spatial attention are applied in parallel at every scale to sharpen endocardial/epicardial boundaries.
- **Transformer bottleneck:** multi-head self-attention provides long-range context at the deepest level, improving delineation under low contrast and complex anatomy.
- **Attention-based explainability:** Grad-CAM/attention visualizations expose decision-relevant regions, supporting transparency and clinical review.

**Loss function.** The final VentrEX-Seg model was optimized using multiclass cross-entropy loss between the predicted voxelwise class probabilities and the reference segmentation labels. The same loss formulation was used throughout training and validation.

### 2.3. Attention-Based Explainability

#### 2.3.1. Modified Attention Grad-CAM

To enhance interpretability, Attention Grad-CAM (AGC) generates attention-weighted heatmaps highlighting critical segmentation regions [17]:

(1) $$L_{\mathrm{AGC}}^{\left(c\right)}=\mathrm{ReLU}\;\left(\sum_{k}\alpha_{k}^{\left(c\right)}\;F_{\mathrm{att}}^{k}\right),$$

where $F_{\mathrm{att}}^{k}$ is the *k*-th attention feature map and $\alpha_{k}^{\left(c\right)}$ is the Grad-CAM weight for class *c*.

#### 2.3.2. Faithfulness Evaluation

The faithfulness of attention mechanisms is validated through perturbation-based tests. These tests, represented here by the violation score, aim to quantify the impact of masking regions identified by the attention maps on the segmentation performance [18]. The violation score $V_{k}$ for class *k* is defined as

(2) $$V_{k}=C_{k}\left(x\right)-C_{k}\left(x_{\mathrm{perturbed}}\right),$$

where $C_{k}\left(x\right)$ represents the confidence score for class *k* given the original input x, and $x_{\mathrm{perturbed}}$ is the input after masking the regions. A positive value of $V_{k}$ indicates that masking these regions reduced the model’s confidence, thereby providing evidence that the identified regions contributed to the prediction.

### 2.4. Automated Papillary-Trabecular Detection

Accurate, automated ventricular quantification requires anatomically faithful separation of blood pool from PM/T. To operationalize this within our cine-CMR workflow, we introduce a slice-wise, adaptive thresholding aid for PM/T that is inspired by threshold-based delineation used in LGE–CMR for hyperenhancement [19] but applied here to cine intensities to support annotation and post-hoc standardization (no LGE is used in the main experiments). The original ACDC masks provide labels for the LV cavity, myocardium, and RV but do not provide a separate annotation class for papillary muscles or trabeculae. In accordance with the ACDC annotation protocol, papillary muscles are included within the LV cavity [14]. For the dedicated PM/T analysis, the post-standardization LV mask produced by the PM/T module was compared with the corresponding manually standardized LV reference mask. Thus, the reported Dice coefficient measures voxel-level agreement under the same PM/T contouring convention.

**Re-annotation workflow:** During expert re-annotation of ACDC cases, the aid proposed candidate PM/T regions inside the LV endocardium and thereby accelerated contouring of $N_{\mathrm{slices}}=288$ short-axis slices. For each slice, the reference $k^{*}$ was obtained by grid search k∈[0.5, 2.5] with step Δk=0.05, maximizing Dice between thresholded candidates and expert PM/T to form

(3) T=μ+k σ,

where μ and σ are the mean and standard deviation of cavity intensities (or a narrow endocardial band). Voxels with intensity >T are flagged as candidate PM/T; light morphology and an optional myocardium constraint yield a provisional PM/T mask. The tool was used only to streamline expert PM/T delineation and to generate training targets for the PM/T standardization stage; it is not an LGE scar detector.

**Features and regression:** Per slice, we extract interpretable descriptors from the LV region $p_{10}/p_{90}$ percentiles, minimum-maximum range, skewness, kurtosis, and contrast and homogeneity (9 features). We rank features using the trained Gradient Boosting Regressor’s impurity-based importance and retain the top four: p90 (90th intensity percentile), contrast, kurtosis, and homogeneity [20]. A Gradient Boosting Regressor predicts the threshold (T); ground-truth *T* values are obtained retrospectively by matching thresholded masks to expert PM/T contours. A gradient-boosting regressor was selected because the PM/T module used a compact set of interpretable intensity and texture descriptors derived from a limited expert-annotated cohort. The model can represent nonlinear relationships between these descriptors and the target threshold while retaining low inference overhead and explicit feature-level inspection. This modular design also avoids introducing an additional data-intensive neural network into the segmentation stage. The selection should therefore be understood as a pragmatic choice for the available annotations and interpretability objectives, rather than as evidence that gradient boosting is universally preferable to end-to-end learning. Hyperparameters were tuned by 5-fold cross-validation on the ACDC subset reviewed by a cardiologist.

**Inference:** At inference time, (1) obtain the LV cavity from VentrEX-Seg; (2) compute features; (3) predict $T_{\mathrm{pred}}$; (4) label voxels $>T_{\mathrm{pred}}$ as candidate PM/T; and (5) apply light morphology to produce the PM/T mask. The resulting masks feed the downstream PM/T standardization module.

PM/T standardization operates at the voxel-label level rather than as a scalar correction of ventricular volume. Before calculating the post-standardization Dice, the predicted and reference LV masks were expressed using the same PM/T contouring convention. Consequently, the standardization procedure modifies the prediction-reference intersection and the numbers of false-positive and false-negative voxels.

### 2.5. Evaluation Metrics

- **95th-percentile Hausdorff distance (HD95):** Let ∂P and ∂G be surfaces of prediction and ground truth and $d\left(x,Y\right)=\min_{y\in Y}{∥x-y∥}_{2}$. We pool bidirectional surface distances {d(p,∂G)}∪{d(g,∂P)} and report the 95th percentile, which is less sensitive to outliers than the maximum Hausdorff distance.
- **Dice Coefficient:** Measures the overlap between the predicted segmentation and the ground truth. It is defined as
  (4) $$\mathrm{Dice}\;\mathrm{Coefficient}=\frac{2\times |A\cap B|}{\left|A|+|B\right|},$$
  where *A* and *B* represent the sets of predicted and ground truth pixels, respectively.
- **Precision:** For each foreground class (*c*), Precision was calculated as
  (5) $${\mathrm{Precision}}_{c}=\frac{{\mathrm{TP}}_{c}+\epsilon }{{\mathrm{TP}}_{c}+{\mathrm{FP}}_{c}+\epsilon },$$
  where ${\mathrm{TP}}_{c}$ and ${\mathrm{FP}}_{c}$ denote the numbers of true-positive and false-positive voxels for class (*c*), respectively, and $\left(\epsilon =10^{-6}\right)$ provides numerical stability. Precision was calculated separately for the RV, myocardium, and LV at the patient level and summarized across the test cohort.
- **Bland-Altman convention:** Differences were computed as Pred - GT (mL) and plotted against the mean (Pred+GT)/2, where Pred and GT refer to the predicted and ground truth masks, respectively; we report mean bias and $\bar{d}\pm 1.96\cdot \mathrm{SD}\left(d\right)$ limits of agreement [21].

## 3. Results

### 3.1. Experimental Setting

We trained VentrEX exclusively on ACDC and followed the official partition, using the provided training, validation, and test subsets. The ACDC cohort was divided at the patient level into 90 training subjects, 10 validation subjects, and 50 held-out test subjects. The test cohort remained unseen during model development. Within the training set, we performed 5-fold patient-level cross-validation for model selection and stability; hyperparameters were chosen based on mean validation loss across folds. The final model was then retrained on the full training set and evaluated once on the held-out labeled test set, which remained unseen during development. Training proceeded for up to 20,000 iterations using the Adam optimizer with a learning rate of $\left(1\times 10^{-4}\right)$, weight decay of $\left(1\times 10^{-4}\right)$, a batch size of 2, and multiclass cross-entropy loss. Early stopping monitored the validation loss with a patience of 1000 iterations and a minimum improvement threshold of $\left(1\times 10^{-4}\right)$. The network depth was four, with residual blocks and instance normalization. The preprocessing and augmentation procedures are detailed in Section 2. Surface-distance metrics were calculated after resampling the prediction and reference masks to an isotropic spacing of 1.25 mm using nearest-neighbor interpolation, whereas volumetric indices and Bland-Altman analyses were calculated using the physical voxel spacing of each evaluation volume. Experiments were conducted on a Microsoft Azure Standard NC16as T4 v3 virtual machine (Microsoft Corporation, Redmond, WA, USA) equipped with an NVIDIA Tesla T4 GPU with 16 GB of memory (NVIDIA Corporation, Santa Clara, CA, USA) and 110 GB of system RAM.

We used patient-level 5-fold cross-validation on the re-annotated subset ($N_{\mathrm{pt}}=20$; $N_{\mathrm{slices}}=288$). Features were z-scored within fold (scaler fit on training folds only) to avoid data leakage. The GBR hyper-parameter grid was as follows: number of estimators ∈{150,200,300}, maximum depth ∈{3,5}, and learning rate ∈{0.05,0.1}; random state = 42. The selected values at the CV optimum were as follows: estimators = 200, depth = 5, learning rate η=0.1. Training minimized mean-squared error loss, and validation metrics (R^2^, RMSE, MAE) were averaged across folds. The held-out ACDC test cohort comprised 50 subjects.

### 3.2. Datasets

#### 3.2.1. Training (Source): ACDC

The Automated Cardiac Diagnosis Challenge (ACDC) provides multicenter, multivendor cine-CMR short-axis studies with expert pixel-level annotations at ED and ES. Labels comprise the LV endocardium, LV epicardium, and RV endocardium. Ground truth was produced under a standardized protocol and adjudicated across sites, enabling benchmarking under realistic inter-center variability [14].

#### 3.2.2. External Target 1: Sunnybrook/LVSC

The Sunnybrook Cardiac MR Left Ventricle Segmentation Challenge (LVSC) [15] contains cine short-axis series with manual LV contours (endocardial and epicardial) delineated by experienced readers under the contest protocol. Because reference labels are restricted to the LV, our external validation on this cohort focuses only on LV cavity and myocardium performance. All 45 Sunnybrook subjects were included in the zero-shot LV evaluation.

#### 3.2.3. External Target 2: MM-WHS (MRI Subset)

The Multi-Modality Whole-Heart Segmentation (MM-WHS) benchmark [16] provides volumetric MRI studies with comprehensive 3D anatomical labels, including the RV, LV, myocardium, atria, and great vessels. We used the MRI subset to assess zero-shot RV performance, while reporting LV and myocardium performance where applicable. The evaluated MM-WHS MRI subset comprised 14 annotated volumes.

Neither Sunnybrook nor MM-WHS provides papillary muscles and trabeculae as a separate reference class. Sunnybrook provides LV endocardial and epicardial contours under its challenge protocol, whereas MM-WHS provides ventricular blood-cavity and LV-myocardial labels. Because the released documentation does not establish that their PM/T assignment conventions match ACDC, we used the external reference masks as distributed. Accordingly, the external analyses measure agreement with each dataset’s released labeling convention, and no external PM/T-specific ground-truth analysis was performed.

### 3.3. Quantitative Evaluation

Table 1 summarizes the patient-level Dice Similarity Coefficient (DSC) and boundary error (HD95, mm) on ACDC. VentrEX achieved the highest ventricular overlap with lower boundary error (RV: DSC 0.9335, HD95 1.13 mm; LV: DSC 0.9541, HD95 0.81 mm), exceeding U-Net/CBAM and matching or surpassing nnU-Net for ventricular structures. For myocardium, nnU-Net yielded the top DSC (0.8399), while VentrEX-Seg remained competitive (0.8259 DSC). Zero-shot generalization was maintained on external cohorts (Table 2)-Sunnybrook LV DSC 0.9053 (HD95 4.95 mm) and MM-WHS RV DSC 0.9236 (HD95 6.61 mm)-indicating robustness across scanner/protocol and label-convention shifts.

The increase in LV Dice from 0.9205 to 0.9541 reflects voxel-level agreement between the LV mask produced after PM/T standardization and the corresponding manually standardized reference mask. PM/T-related voxels at the blood-pool/endocardial interface were reassigned according to the same contouring convention in the prediction and reference. Consequently, the prediction–reference intersection and the numbers of false-positive and false-negative voxels changed; the result is not attributable to a scalar adjustment of ventricular volume.

In addition to segmentation accuracy, we evaluated the computational requirements of the neural segmentation models under a standardized inference protocol. As reported in Supplementary Table S1, VentrEX-Seg contained 10.85 million trainable parameters, approximately 33.3% fewer than CBAM and 33.0% fewer than nnU-Net. Compared with CBAM, VentrEX-Seg reduced mean inference latency by 67.5% and peak allocated GPU memory by 34.2%. Relative to the nnU-Net, VentrEX-Seg demonstrated comparable inference latency (85.75 versus 83.49 ms/case) while requiring 28.3% less peak GPU memory.

**Figure 3:**
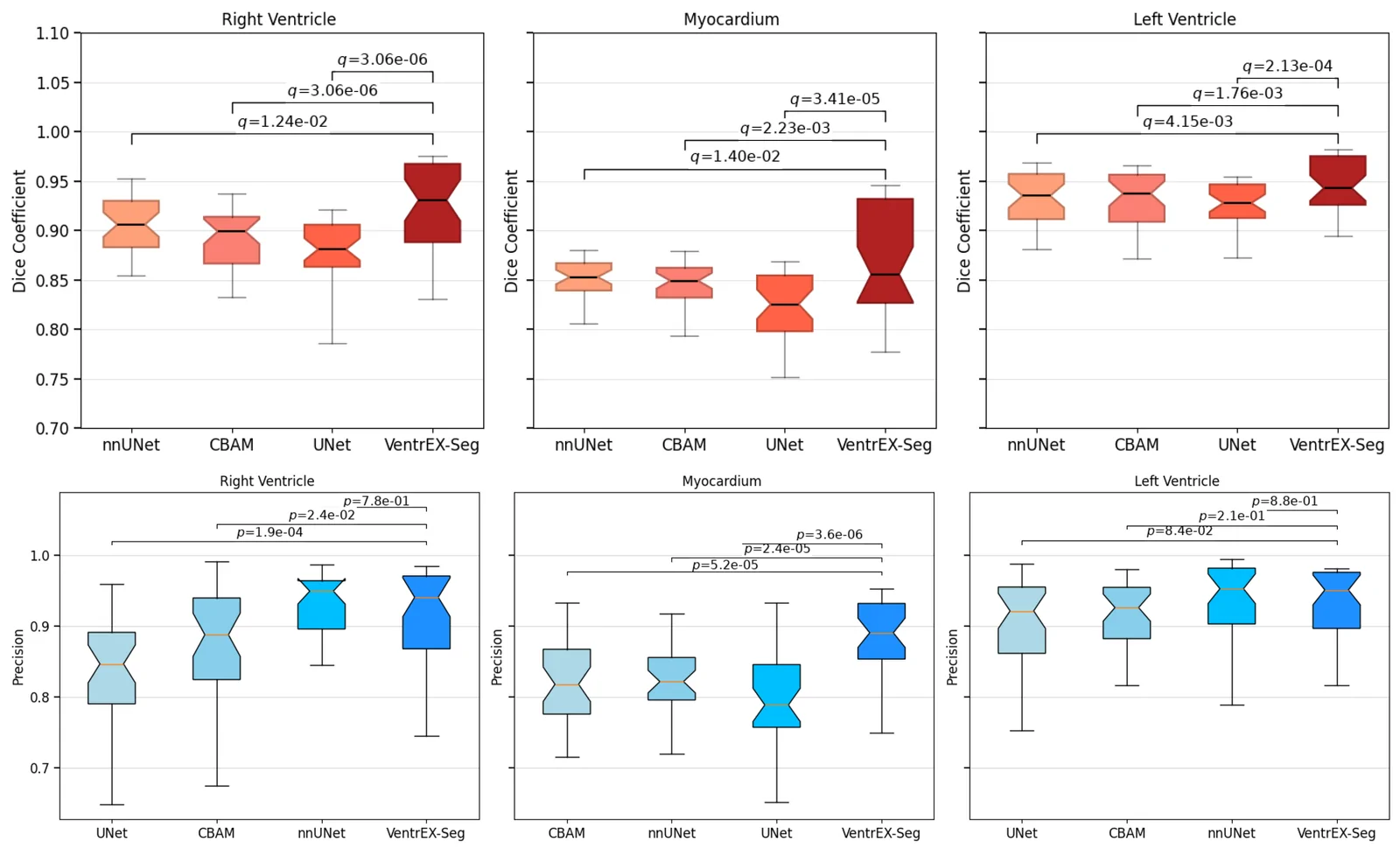
Patient-level Dice and Precision distributions across models. The upper row shows Dice coefficients, and the lower row shows Precision for the RV, myocardium, and LV. Boxplots represent the patient-level distributions for U-Net, CBAM, nnU-Net, and VentrEX-Seg. Pairwise Wilcoxon signed-rank comparisons are overlaid; (*p* < 0.05) indicates statistical significance.

**Statistical significance.** Figure 3 presents patient-level distributions of Dice in the upper row and Precision in the lower row for the RV, myocardium, and LV. Precision complements Dice by quantifying the proportion of predicted foreground voxels that agree with the reference segmentation, thereby reflecting false-positive segmentation burden. Paired Wilcoxon tests indicated significant gains for VentrEX-Seg vs. U-Net and CBAM on RV (both $p=2.0\;\times \;10^{-3}$) and on LV vs. U-Net ($p=3.7\;\times \;10^{-2}$) and CBAM ($p=4.9\;\times \;10^{-2}$); differences vs. nnU-Net were not significant for RV/LV. For myocardium, the differences relative to nnU-Net and CBAM were statistically significant; however, VentrEX-Seg achieved a lower mean Dice coefficient than both models, consistent with the structure-specific performance reported in Table 1.

**Figure 4:**
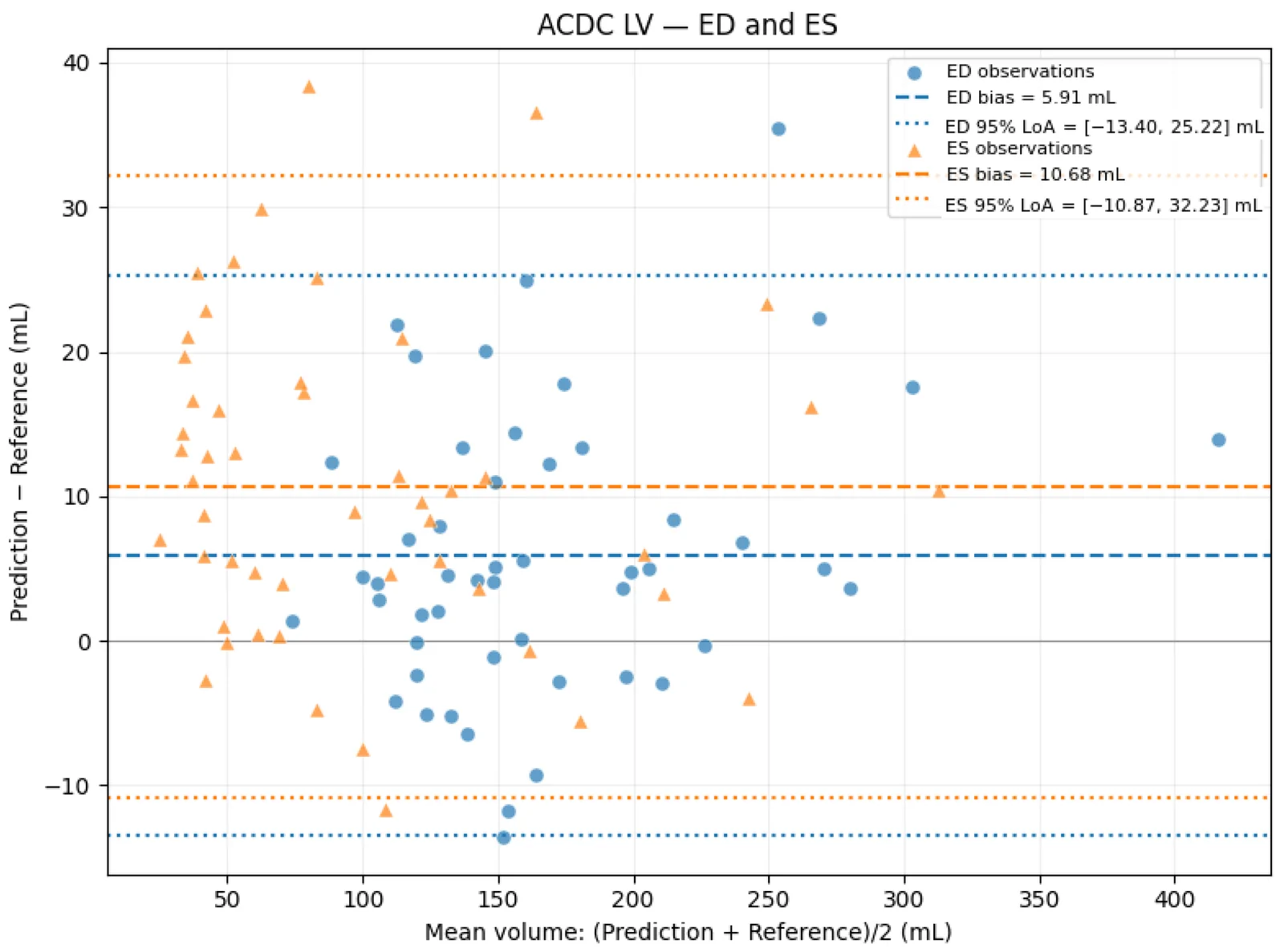
Bland–Altman analysis of LV volumes at ED and ES for the 50-patient held-out ACDC test cohort. Blue circles represent ED observations, and orange triangles represent ES observations. Differences were defined as prediction minus reference. Dashed lines indicate phase-specific mean biases, and dotted lines indicate the corresponding 95% limits of agreement calculated as bias ±1.96 SD.

**Figure 5:**
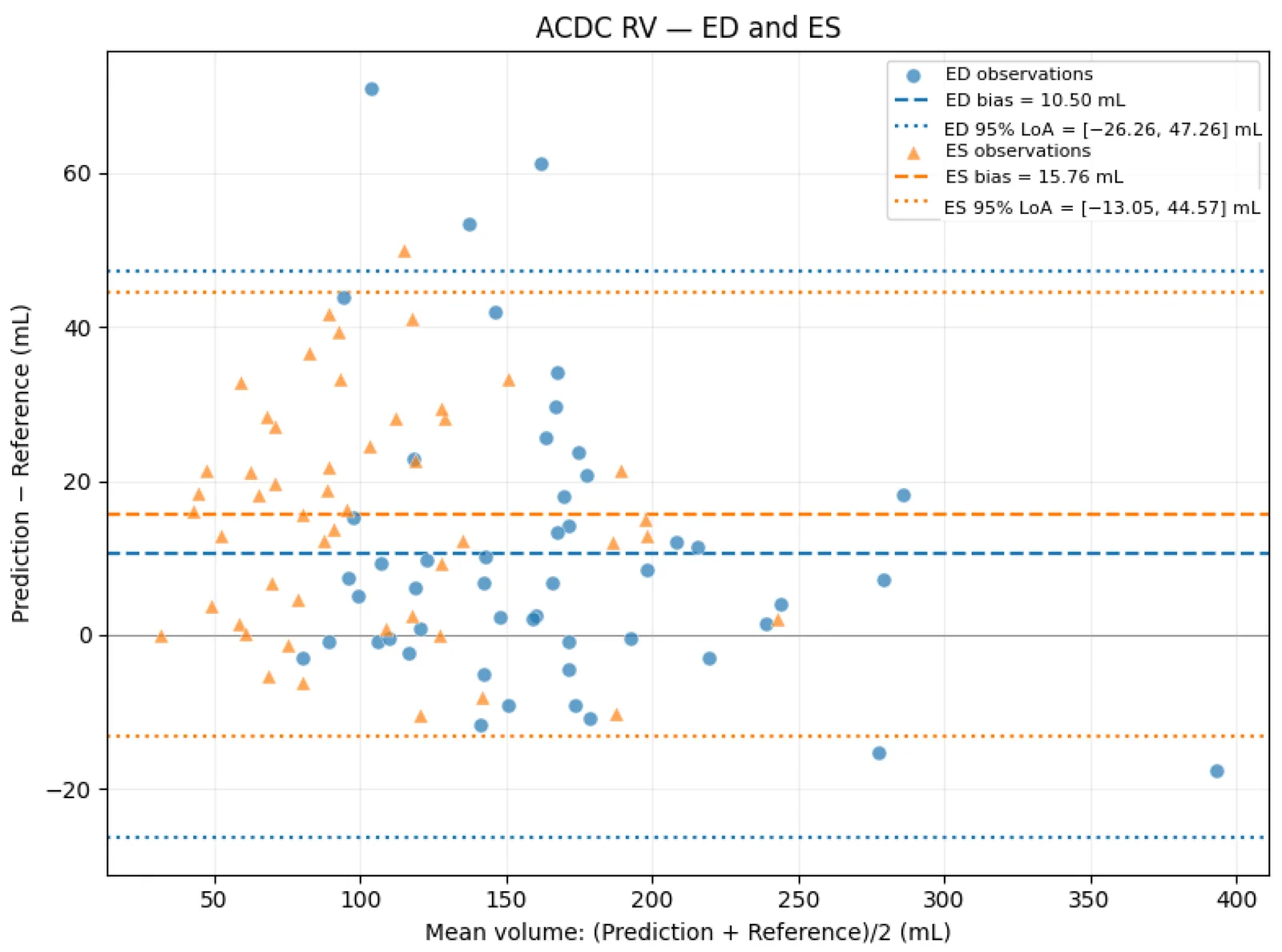
Bland–Altman analysis of RV volumes at ED and ES for the 50-patient held-out ACDC test cohort. Blue circles represent ED observations and orange triangles represent ES observations. Differences were defined as prediction minus reference. Dashed lines indicate phase-specific mean biases, and dotted lines indicate the corresponding 95% limits of agreement calculated as bias ±1.96 SD.

**Figure 6:**
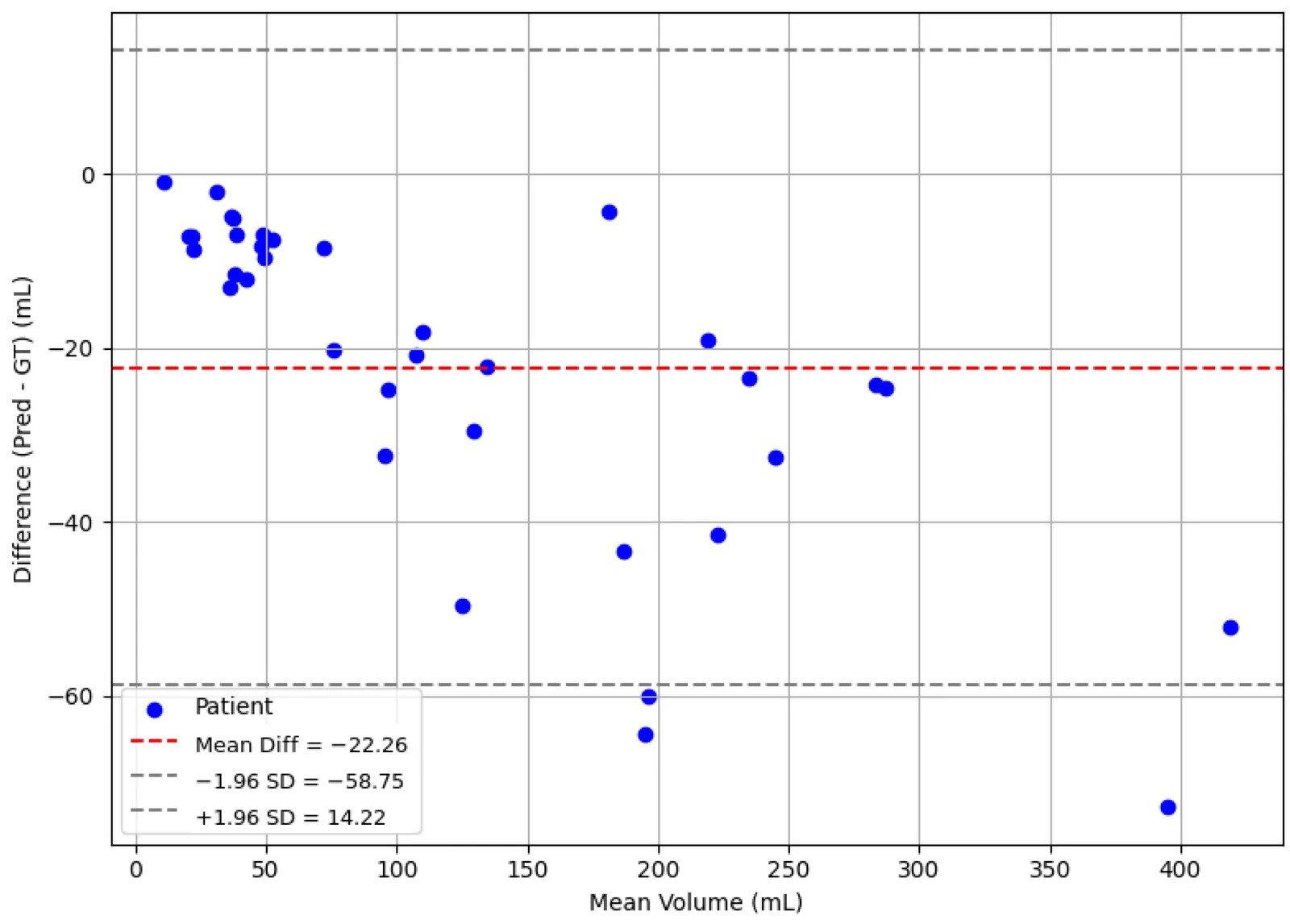
Sunnybrook LV (pooled). External LV volumes exhibit a larger negative bias (underestimation) and wider limits, reflecting cross-dataset differences in contour conventions and acquisition protocols.

**Figure 7:**
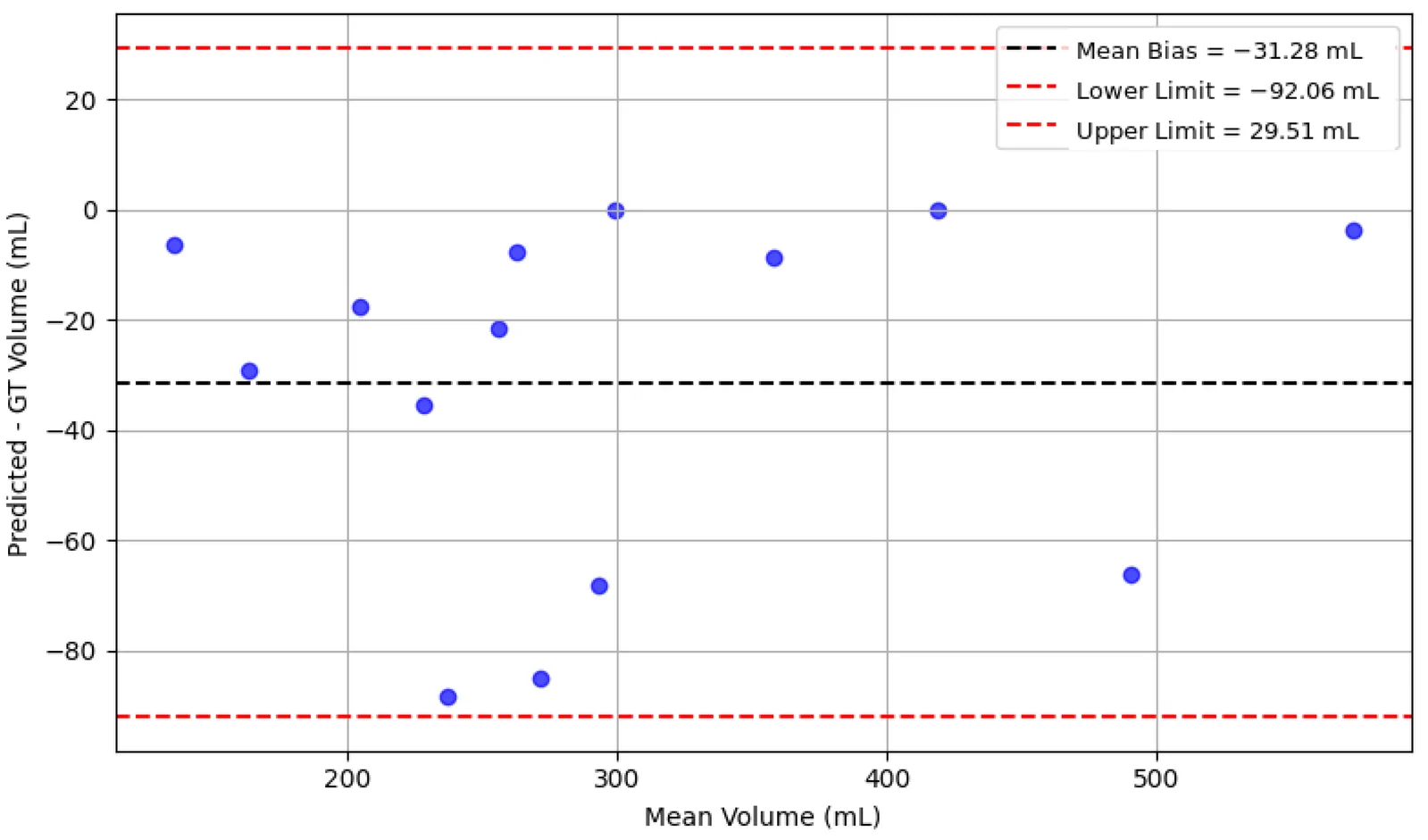
MM-WHS RV (pooled). External RV shows a comparable negative bias and wider limits.

**Bland-Altman analysis.** Bland-Altman analysis was performed using the direct VentrEX-Seg predictions from all 50 patients in the held-out ACDC test cohort. For the LV (Figure 4), the mean prediction-reference bias was 5.91 mL at ED, with 95% limits of agreement from −13.40 to 25.22 mL, and 10.68 mL at ES, with limits from −10.87 to 32.23 mL. For the RV (Figure 5), the corresponding bias was 10.50 mL at ED, with limits from −26.26 to 47.26 mL, and 15.76 mL at ES, with limits from −13.05 to 44.57 mL. In the external zero-shot analyses, the Sunnybrook LV analysis (Figure 6) yielded a bias of −22.26 mL, with 95% limits of agreement from −58.75 to 14.22 mL. The MM-WHS RV analysis (Figure 7) yielded a bias of −31.28 mL, with limits from −92.06 to 29.51 mL.

### 3.4. Explainability Analysis

**Figure 8:**
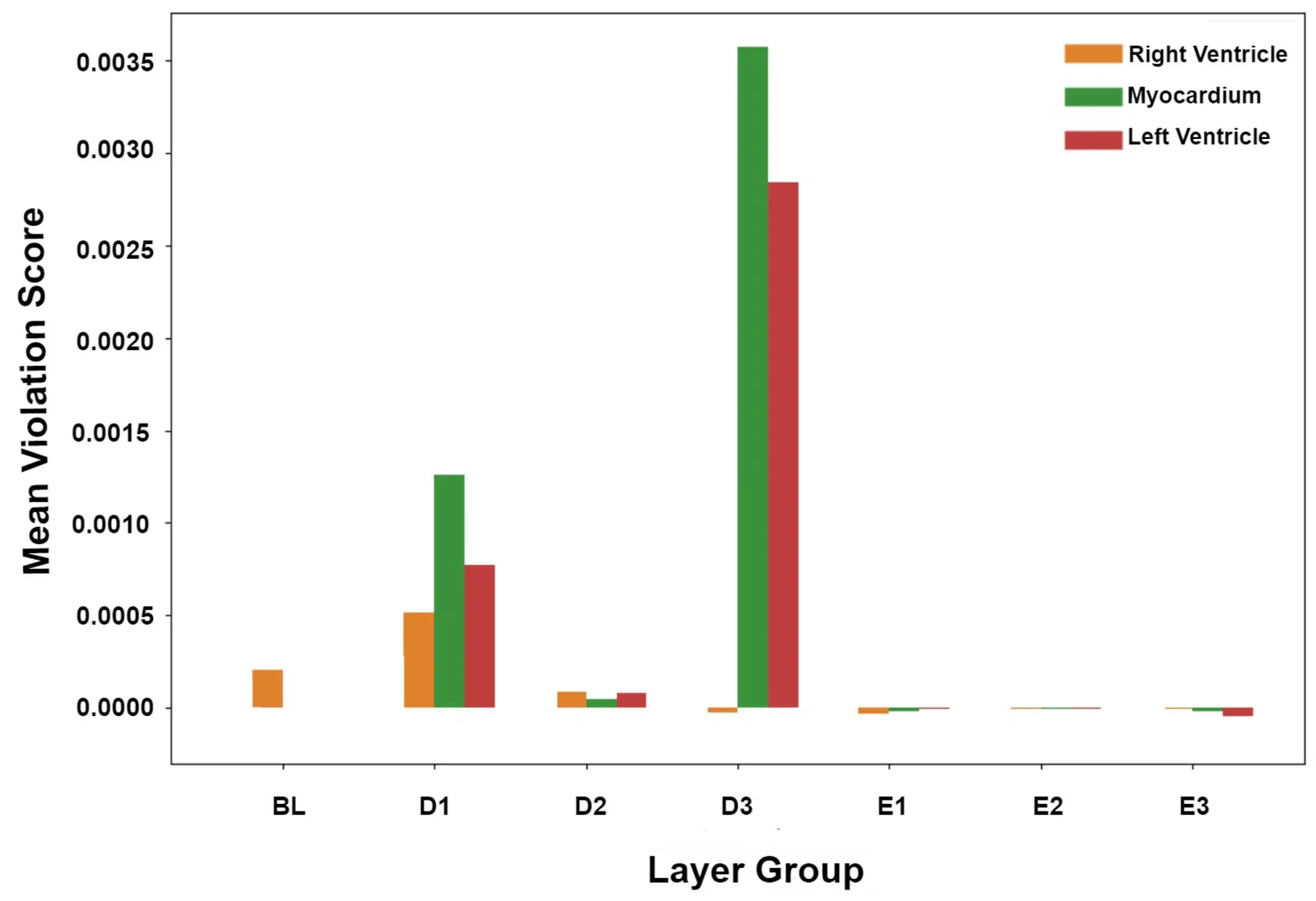
Perturbation sensitivity by layer group. Violation scores quantify performance degradation after masking attention-identified regions. Encoder D3 and the bottleneck (BL) are most sensitive for LV/MYO, whereas decoder stages show progressively stabilized saliency.

**Figure 9:**
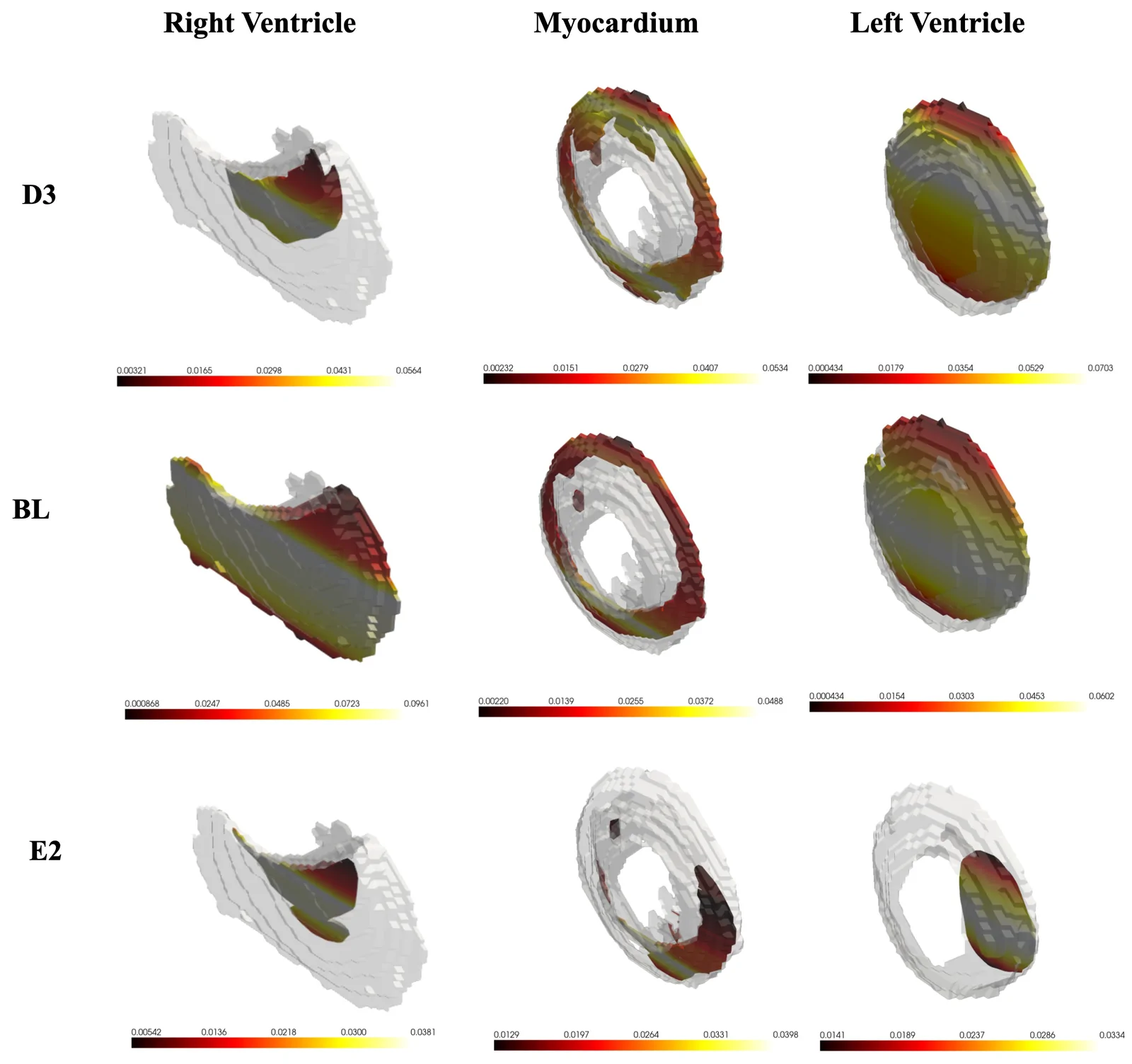
3D attention maps (AGC) across classes and layer groups. Attention-weighted activations localize to anatomical borders and are refined from encoder (D3) to bottleneck (BL) to decoder (E2). Darker regions denote higher attention weights.

Perturbation sensitivity (Figure 8) identified the D3 downsampling and bottleneck layers as most influential for LV/MYO segmentation, while decoder layers exhibited stabilized saliency. 3D Attention Grad-CAM (Figure 9) localized high-importance regions to endocardial/epicardial borders with progressive refinement from encoder to decoder. Across held-out folds, threshold prediction achieved $R^{2}=0.825\pm 0.09$ and ${\mathrm{MAE}}_{T}=5.02\pm 0.54$ grayscale intensity units on the intensity scale used by the PM/T module. Here, T=μ+kσ; therefore, the reported MAE refers to the predicted absolute threshold *T*, not to the bounded dimensionless multiplier *k*.

### 3.5. Qualitative Analysis and Clinical Assessment

**Figure 10:**
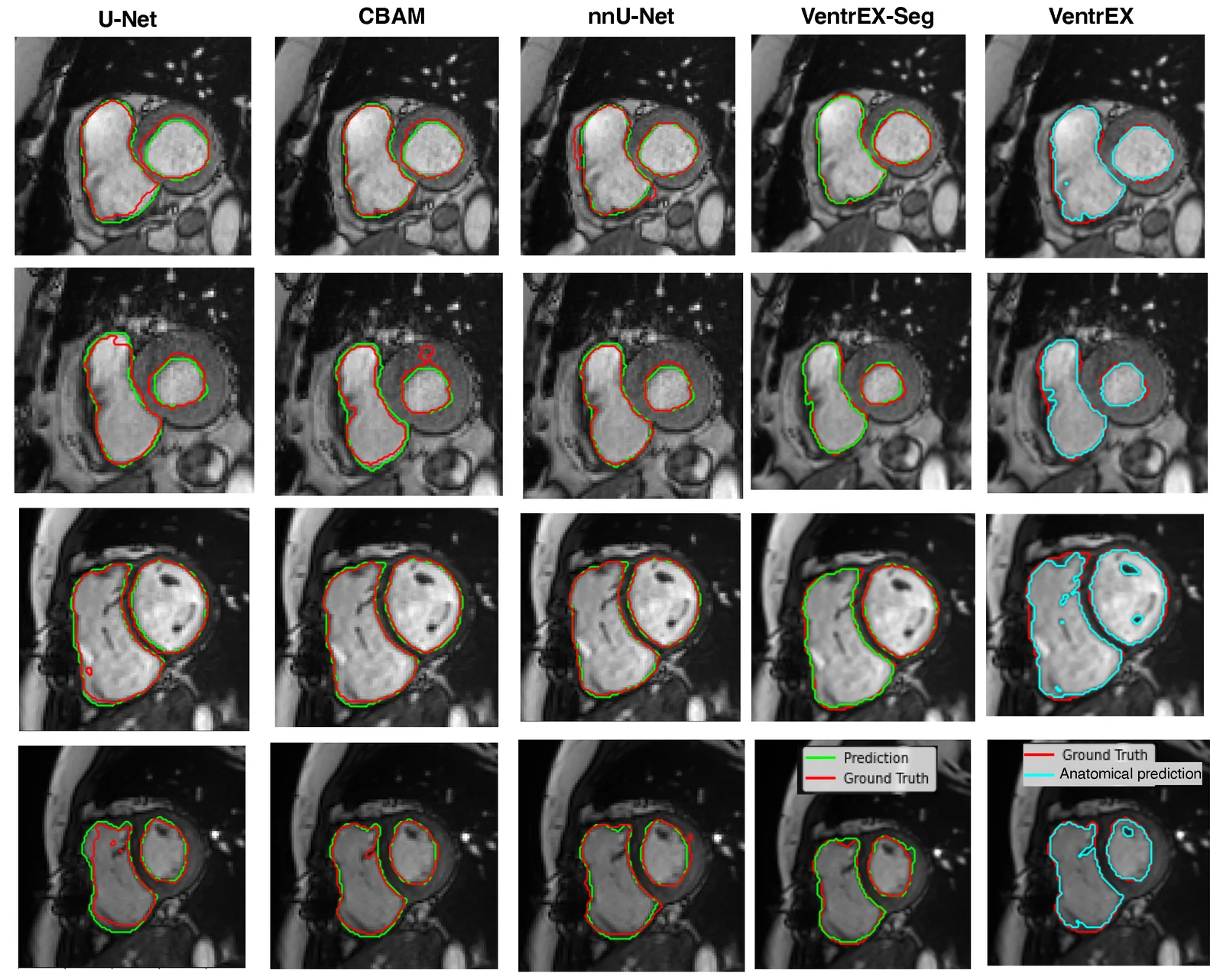
Representative 2D overlays on ACDC. Qualitative comparison among U-Net, CBAM, nnU-Net, VentrEX-Seg, and VentrEX. Green contours denote reference; red contours denote predictions. VentrEX reduces papillary-muscle and trabecular “leaks” and yields crisper endocardial borders across ED/ES.

**Figure 11:**
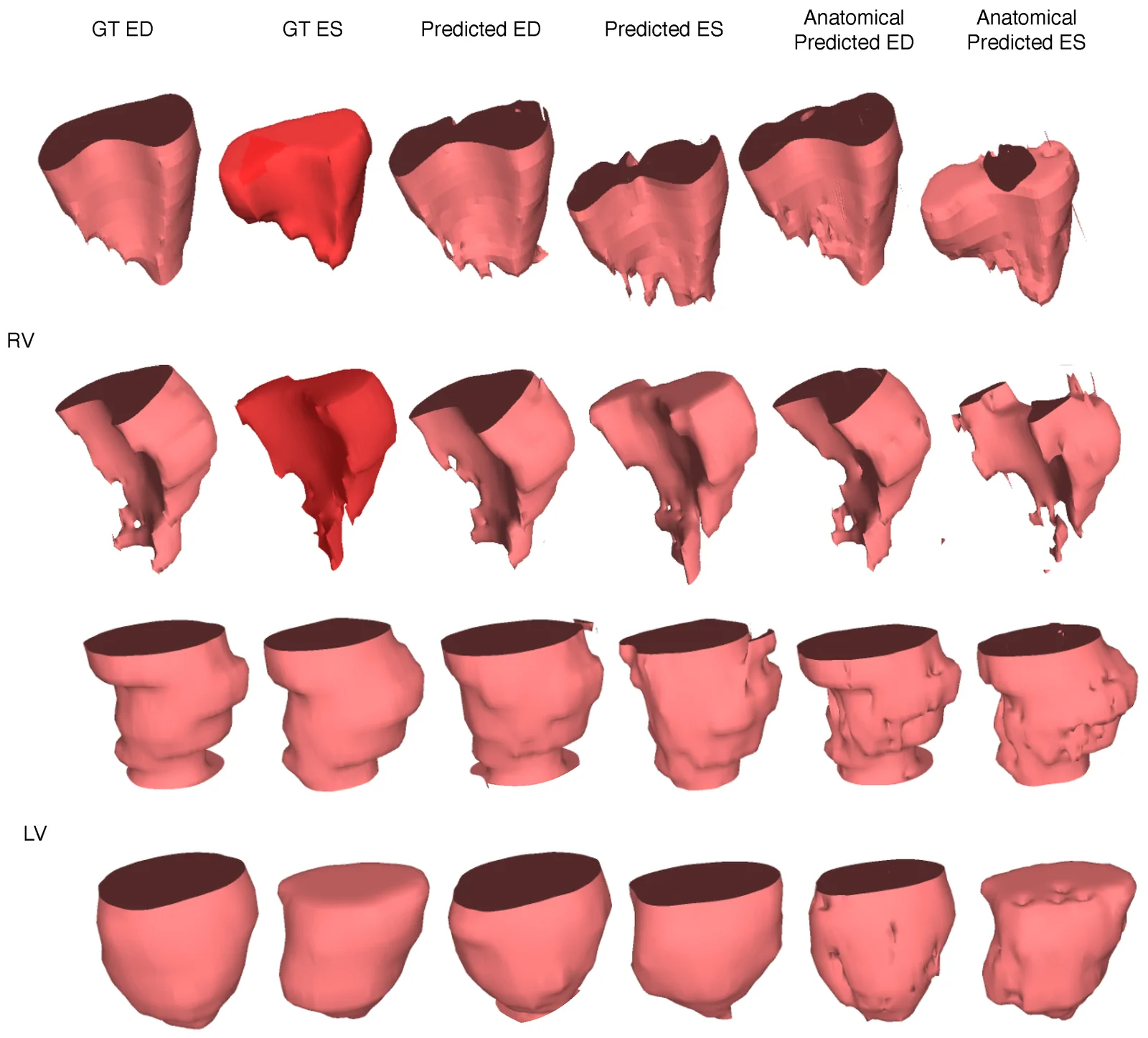
LV 3D reconstructions across ED/ES (ACDC). Columns show ground truth, direct predictions, and anatomical predictions after PM/T standardization. Removing papillary/trabecular signal from the LV cavity reduces systematic overestimation and produces anatomically consistent endocardial surfaces.

**Figure 12:**
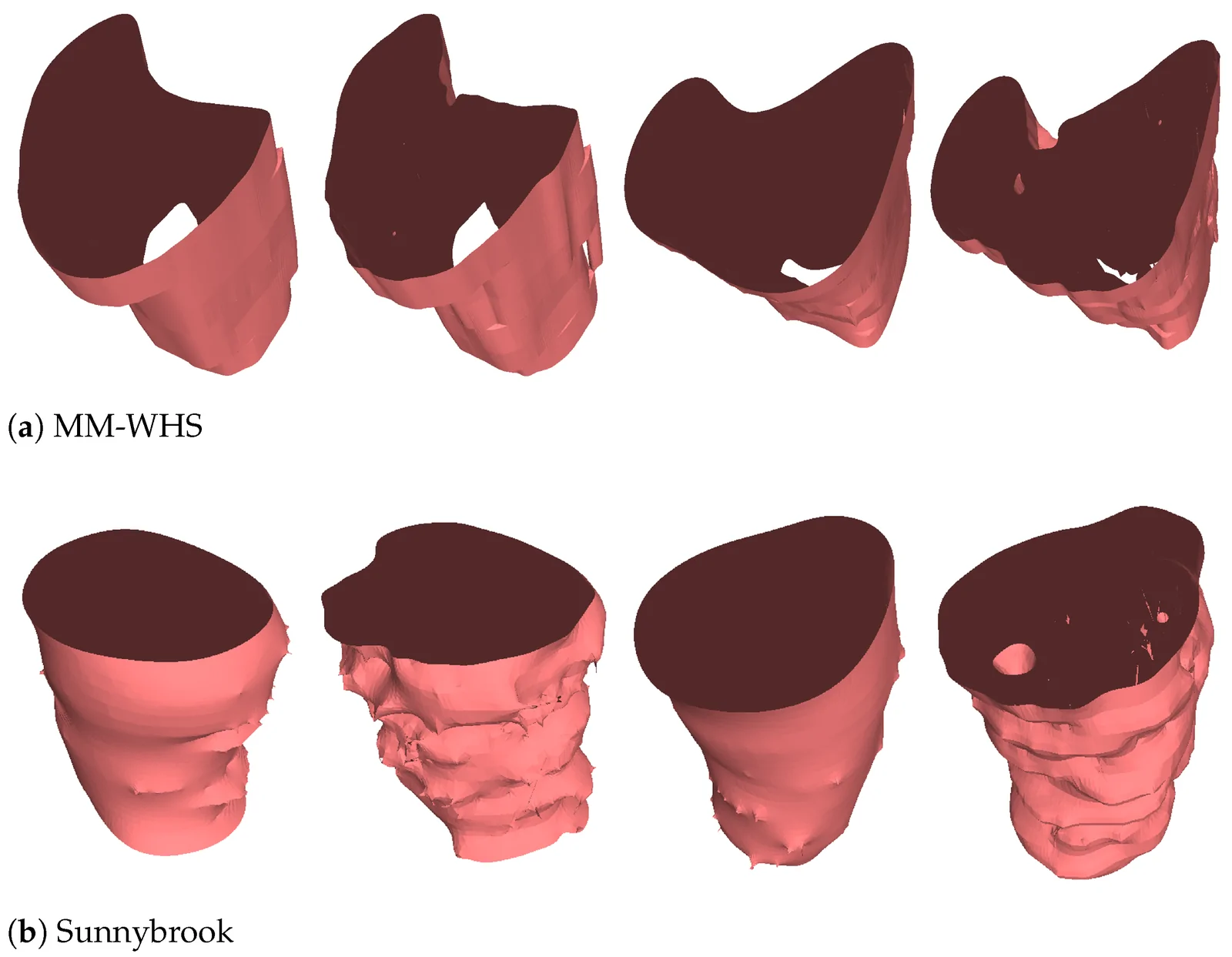
External 3D ventricular surfaces. Results are shown for (**a**) MM-WHS and (**b**) Sunnybrook. The first and third columns show the direct VentrEX-Seg outputs, whereas the second and fourth columns show the final VentrEX outputs after PM/T standardization. The standardized outputs exhibit improved basal/apical continuity and fewer spurious bridges.

Representative overlays (Figure 10) show VentrEX yields crisper endocardial borders with fewer papillary/trabecular “leaks” than baselines. 3D reconstructions (Figure 11) illustrate that PM/T-standardization reduces cavity overestimation and enforces anatomically plausible LV surfaces at ED/ES. External 3D surfaces (Figure 12) preserve basal and apical continuity and suppress spurious bridges, consistent with improved boundary metrics.

## 4. Discussion

Quantitative evaluation showed that VentrEX achieved the strongest performance for the principal ventricular cavity endpoints, with improved Dice and HD95 for both the LV and RV. These gains support the intended design of the framework, which combines parallel channel-spatial attention and a Transformer bottleneck with anatomical PM/T standardization. For myocardium, VentrEX-Seg remained competitive, although nnU-Net achieved a modestly higher Dice coefficient (0.8399 versus 0.8259). This difference may reflect the challenges associated with the myocardium’s thin geometry, partial-volume effects, and the simultaneous delineation of endocardial and epicardial boundaries. Moreover, the PM/T standardization stage targets ventricular blood-pool boundaries and does not directly refine the myocardial output. Accordingly, VentrEX’s principal contribution lies in ventricular segmentation and convention-consistent volumetry rather than uniform improvement across every anatomical structure.

The statistical analysis further underscores VentrEX’s robustness in identifying subtle anatomical features, translating into fewer boundary errors and tighter dispersion. This matters clinically: precise anatomy reduces measurement noise and improves repeatability, which directly benefits downstream assessment and decision-making. In parallel, Grad-CAM and perturbation-based tests increased transparency by localizing decision-relevant regions to anatomical borders, addressing the “black-box” concern and supporting clinician trust.

Qualitatively, VentrEX consistently delineated PM/T, a prerequisite for reliable volumetry. The 3D visualizations showed that relabeling PM/T according to the selected contouring convention via our PM/T stage avoids systematic overestimation and yields more anatomically plausible endocardial surfaces across ED/ES. Temporal examples further indicated that the model resists mid-systolic overestimation, where thin myocardial walls and papillary bundles often confound segmenters. The objective of PM/T standardization is to reduce convention-dependent variation in measured ventricular volumes and myocardial mass. The observed changes in segmentation agreement and volumetric bias should not be interpreted as evidence that PM/T assignment changes intrinsic ventricular systolic function or independently determines the cardiac phenotype.

Finally, comparisons of 3D geometries in cardiomyopathy cases illustrated the clinical impact of anatomical fidelity. Accurate treatment of PM/T substantially influences LV volume and LVEF estimation, which are key biomarkers for diagnosing and staging cardiomyopathies. In our cohort, the PM/T stage provides convention-consistent voxel labeling at the blood-pool/endocardial interface. This anatomical standardization is expected to translate to more stable LVEF estimates. Several limitations should be acknowledged. The current framework remains sensitive to the bottleneck configuration and does not fully capture the range of contouring conventions, acquisition variability, and pathological presentations encountered in clinical practice. In particular, the PM/T regressor may be less reliable in extreme pathologies that substantially alter myocardial or blood-pool intensity distributions. Severe hypertrophy, extensive tissue heterogeneity, flow-related artifacts, or unusually poor blood-myocardium contrast could shift the regressor features beyond the distribution represented by the current expert-annotated cohort. In such cases, the predicted threshold may not reliably separate PM/T signal from the blood pool. Future work should therefore include prospective multicenter and pathology-stratified external validation, expansion of the expert annotations, uncertainty-based detection of cases requiring manual review, further stabilization of the Transformer bottleneck, and broader harmonization of contouring conventions.

## 5. Conclusions

We presented VentrEX, an anatomically guided pipeline for cine-CMR ventricular analysis that couples a 3D encoder-decoder with parallel channel-spatial attention and a Transformer bottleneck (VentrEX-Seg) and adds an automated PM/T stage for volumetric standardization. Trained exclusively on ACDC and evaluated zero-shot on Sunnybrook (LV) and MM-WHS MRI (RV), the pipeline delivered higher Dice and lower boundary error than strong baselines and reduced volumetric bias in Bland-Altman analyses. Qualitative 2D/3D results and attention-based visualizations showed anatomically plausible borders, fewer PM/T leaks, and improved transparency for clinical review.

This work addresses two persistent obstacles to clinical translation, domain shift and PM/T-driven volume errors, by combining global context (Transformer), fine-scale attention, and explicit PM/T handling. Future efforts will target prospective multi-center validation, uncertainty quantification, deeper stabilization of the Transformer block, extension to additional cardiac structures and temporal modeling, and broader harmonization of contour conventions. We will also investigate extending VentrEX to cardiac CT through modality-specific adaptation and validation, with potential applications in convention-consistent ventricular volumetry and multimodal cardiac assessment. Collectively, these directions aim to further improve the reproducibility and clinical readiness of automated cardiac segmentation.

## Figures and Tables

**Table 1:** Segmentation accuracy on the held-out ACDC test cohort (N=50 subjects). Patient-level Dice coefficient (DSC) and HD95 (mm) are reported for the RV, myocardium, and LV across models. VentrEX attains the highest ventricular overlap and lower boundary error; nnU-Net has the best MYO DSC. Values are reported at the patient level; myocardium for VentrEX was not computed in this run (cavity endpoints targeted). (*n/a*: not computed for this run; we restrict VentrEX endpoints to cavity structures (RV/LV)).

Model	RV		MYO		LV	
	DSC	HD95	DSC	HD95	DSC	HD95
U-Net	0.8241	2.31	0.7201	6.20	0.8513	6.74
CBAM	0.8605	3.49	0.8289	1.69	0.9124	1.70
nnU-Net	0.8788	1.38	**0.8399**	1.78	0.9246	0.87
VentrEX-Seg	0.8696	1.31	0.8259	2.34	0.9205	0.89
**VentrEX**	**0.9335**	1.13	*n/a*	*n/a*	**0.9541**	0.81

**Table 2:** Zero-shot external generalization on Sunnybrook (N=45 subjects) and MM-WHS MRI (N=14 annotated volumes). Dice and HD95 (mm) are reported for the available target structures without external fine-tuning. VentrEX maintains strong performance without fine-tuning, indicating robustness to domain shift and label-convention differences.

Dataset	Struct.	VentrEX		VentrEX-Seg	
		Dice	HD95	Dice	HD95
Sunnybrook (LVSC)	LV	0.9053	4.95	0.8194	7.99
MM-WHS (MRI)	RV	0.9236	6.61	0.8251	12.69

## Data Availability

The datasets analyzed during the current study are publicly available and can be accessed as follows: ACDC (Automated Cardiac Diagnosis Challenge): https://www.creatis.insa-lyon.fr/Challenge/acdc/ (accessed on 31 January 2025); Sunnybrook Cardiac MR Left Ventricle Segmentation Challenge (LVSC): https://www.cardiacatlas.org/challenges/lv-segmentation-challenge/ (accessed on 12 March 2025); and MM-WHS (Multi-Modality Whole-Heart Segmentation Challenge): https://zmiclab.github.io/zxh/0/mmwhs/ (accessed on 20 March 2025). All datasets are de-identified and publicly available subject to the terms of their respective repositories. No new data were generated for this study. The source code, pretrained model weights, environment specification, and instructions for running the complete VentrEX pipeline are publicly available at https://github.com/ablabedoui1/VENTREX (accessed on 15 August 2026).

## Supplementary Materials

The following supporting information can be downloaded at https://www.mdpi.com/article/10.3390/jimaging12090416/s1. Table S1: Retrospective computational-efficiency benchmark on the 50-patient ACDC test set. All models were evaluated using FP32 precision, a batch size of 1, an NVIDIA Tesla T4 GPU, and 10 warm-up iterations. Inference latency includes the forward pass and argmax label decoding but excludes data loading and preprocessing. Values are reported as mean ± standard deviation across patients.

## Abbreviations

ACDC	Automated Cardiac Diagnosis Challenge
AGC	Attention Grad-CAM
CBAM	Convolutional Block Attention Module
CMR	Cardiac Magnetic Resonance
DSC	Dice Similarity Coefficient
ED/ES	End-diastole/End-systole
HD95	95th-percentile Hausdorff distance
LR	Learning rate
LV	Left ventricle
LVEF	Left ventricular ejection fraction
LVM	Left ventricular mass
LVSC	Left Ventricle Segmentation Challenge (Sunnybrook)
MM-WHS	Multi-Modality Whole-Heart Segmentation
MYO	Myocardium
PM/T	Papillary muscles and trabeculae
ReLU	Rectified Linear Unit
RV	Right ventricle
VentrEX-Seg	Core 3D segmentation module of the VentrEX pipeline
