# Supplementary material for: VentrEX: An Anatomically Guided Deep Learning Pipeline for Ventricular Segmentation in Cine Cardiac MRI
Source: J Imaging. 2026 Sep 3;12(9):416. doi: 10.3390/jimaging12090416 (PMC13608386; doi:10.3390/jimaging12090416)
Supplement: Supplementary file 1 [file jimaging-12-00416-s001.zip › jimaging-4444883-SM.pdf]

Supplementary Materials: VentrEX: An Anatomically Guided Deep Learning Pipeline for Ventricular Segmentation in Cine Cardiac MRI

Abla Bedoui [1,*], Julieta Anahí Rancati [2] , Ignacio Lugones [2] and Mohammed Cherkaoui [1]

Table S1. Retrospective computational-efficiency benchmark on the 50-patient ACDC test set. All models were evaluated using FP32 precision, a batch size of 1, an NVIDIA Tesla T4 GPU, and 10 warm-up iterations. Inference latency includes the forward pass and argmax label decoding but excludes data loading and preprocessing. Values are reported as mean ± standard deviation across patients.

Model	Trainable parameters	FP32 parameter footprint (MB)	Checkpoint size (MB)	Inference latency (ms/case)	Peak allocated GPU memory (MB)
CBAM	16,278,386	65.114	195.457	263.865 ± 22.587	551.089
nnU-Net	16,190,436	64.762	194.323	83.487 ± 6.156	506.258
VentrEX-Seg	10,850,818	43.403	130.348	85.750 ± 6.297	362.819

All models received an input tensor of $(1 \times 1 \times 16 \times 144 \times 144)$, including the batch and channel dimensions. The FP32 parameter footprint was calculated as four bytes per trainable parameter. Checkpoint files include additional saved training states and therefore exceed the theoretical parameter footprint. Peak GPU memory denotes the maximum memory allocated by PyTorch during inference. The reported latency values concern the neural segmentation stage and do not include PM/T standardization.
